# Trajectories of Child and Caregiver Positive Coping Following a Brief Emotion‐Focused Family Therapy (EFFT) Intervention

**DOI:** 10.1111/jmft.70029

**Published:** 2025-05-08

**Authors:** Imogen M. Sloss, Jackson Smith, Laura Colucci, Mirisse Foroughe, Dillon T. Browne

**Affiliations:** ^1^ Department of Psychology University of Waterloo Waterloo Ontario Canada; ^2^ Centre for Mental Health Research and Treatment Waterloo Ontario Canada; ^3^ Family Psychology Centre Toronto Ontario Canada

**Keywords:** emotion‐focused family therapy, family functioning, multilevel modelling, positive coping, social support

## Abstract

Families play an influential role in promoting positive coping (PC) among youth, which has led to the development of family‐based interventions, such as emotion‐focused family therapy (EFFT). The present study examined trajectories of PC over 1 year following a 2‐day virtual caregiver group EFFT intervention. Participants included 155 caregivers who attended the EFFT intervention. Caregivers completed measures on themselves and up to four children at six time points from pre‐intervention to 12‐month follow‐up. Higher‐order growth curve analysis modelled trajectories of PC for individuals nested within families. Participants exhibited an increase in PC over 12 months. Caregivers had higher initial PC levels than children and improved at a slower rate. Finally, participants in families with higher social support and lower family dysfunction had higher baseline PC. These variables did not predict change. Findings reveal that aspects of the family environment are related to PC, highlighting the importance of family‐based interventions.

Families, and especially parents, are critical resources in the promotion of positive coping (PC) in children (Clark et al. 2002; Ren et al. 2018). This principle lies at the center of family‐based therapeutic intervention for children with mental health problems and their caregivers. Emotion‐focused family therapy (EFFT) is an approach that involves family members in treatment to support youth with various mental health challenges (Foroughe et al. 2019). EFFT has been adapted into a 2‐day caregiver group intervention, and this format is associated with positive changes in child psychopathology and parental self‐efficacy over 12 months (Foroughe et al. 2023). It remains unclear if similar patterns are observed in terms of PC, including the extent to which changes operate at the family‐ versus individual‐level and what moderators affect these trajectories. Moreover, the virtual (telehealth) adaptation of EFFT groups—developed during the COVID‐19 pandemic—has not been sufficiently studied (Foroughe et al. 2019). Thus, we investigated whether child and caregiver PC changes in the year following a brief virtual caregiver EFFT intervention. Furthermore, we explored the factors that predict how PC changes over time, with particular attention paid to caregiver social support and family dysfunction.

## 1 Positive Coping Within Family Systems

In response to stressors, families cope in varying ways, some of which are more adaptive than others. Research suggests that active coping strategies (e.g., problem‐focused coping, social‐support seeking, and positive reappraisals) are associated with a variety of positive outcomes, such as higher well‐being and psychological life quality, and lower stress, depressive symptoms, anxiety, and insomnia (Budimir et al. 2021; Perez‐Tejada et al. 2019). Active strategies involve taking control of the situation to regulate the stressor (Perez‐Tejada et al. 2019). In contrast, passive coping strategies, such as avoidance, involve distancing oneself from the stressor, preventing individuals from responding effectively to the situation (Perez‐Tejada et al. 2019).

Given that coping strategies are associated with various psychological outcomes, it is important to examine the factors that promote or hinder PC. Using a multilevel design, Brincks et al. (2010) found that a significant proportion of variance in coping was attributed to the family and individual level. Therefore, family‐level environmental factors should be considered in relation to PC. The Double ABC‐X Model explores how families respond to and cope with stressors (McCubbin and Patterson 1983). This model posits that positive family coping and adaptation result from the interaction between the stressor, family resources (e.g., social support, functional family processes), and the family's perception of the stressor. Furthermore, this model considers how stressors can pile up and how families may need to expand on their existing resources (e.g., seeking out professional services) to respond resiliently to the presenting challenges. In the Family Stress Model, Masarik and Conger (2017) explore stressors' cascading effect on the family system and how stressors can impact dyadic relationships and individual members within the family. However, they present various risk and protective factors that can influence this progression.

Functional family processes and social support are two resources, or protective factors, that have been explored in relation to PC. Researchers have found that positive family functioning is related to higher active coping (e.g., problem‐solving, cognitive restructuring, and seeking social support) and lower avoidance and emotion‐focused coping for children and caregivers (Cowen et al. 1990; Crowe and Lyness 2014; Kritzas and Grobler 2005; Mutimer et al. 2007; Ren et al. 2018; Tschann et al. 1996). Social support is also associated with PC (Cowen et al. 1990; Mutimer et al. 2007; Tian et al. 2021). One mechanism through which family functioning and social support are associated with caregiver PC is through reducing stress and mental health challenges (Ghazanfar and Shafiq 2016; Thoits 1995). Support, both within and outside of the family, often involves emotional (e.g., encouragement and advice) and practical (e.g., provision of physical resources) assistance, which can increase an individual's ability to cope effectively (DeLongis and Holtzman 2005; Thoits 1995).

Additionally, lower stress and mental health challenges are associated with positive parenting and parent–child relationships (Risi et al. 2021), which may, in part, explain the association between family resources (family functioning and caregiver social support) and PC in children. Caregivers who are authoritative in their parenting have more consistent parent–child interactions, engage in higher parental monitoring and limit‐setting, and have children with higher PC (Clark et al. 2002; Cowen et al. 1990; Kritzas and Grobler 2005). When relationships are healthier, children have the opportunity to learn how to problem‐solve, communicate, and regulate their emotions in productive ways (Ren et al. 2018). For example, children who confide in their parents and are met with acceptance are more likely to engage in problem‐focused coping because their parents can guide them in how to approach the issue in an effective way (Clark et al. 2002). Lastly, children learn how to respond to stressors by modelling the behavior of those around them, including their caregivers (Clark et al. 2002; Ren et al. 2018). More research is needed to improve our understanding of the longitudinal interplay between the family environment and PC, including identifying trajectories in clinical settings, and examining how PC operates at different levels of family organization.

## 2 Emotion‐Focused Family Therapy (EFFT)

EFFT is a family‐based approach to supporting youth mental health that emerged out of Emotion Focused Therapy (EFT) and family systems theory. It was originally implemented in eating disorder treatment (LaFrance‐Robinson et al. 2016). The last decade has seen remarkable growth in interest in the EFFT model as a transdiagnostic approach. It has been manualized and consists of four modules, as described by Foroughe et al. (2019) and LaFrance‐Robinson et al. (2015). First, parents are taught *emotion coaching*, which involves acknowledging and validating their child's primary emotions and meeting their corresponding needs. Second, EFFT helps family members redress historical harms or psychological injuries through the facilitation of *therapeutic apologies*. Third, parents are guided to address their *emotional blocks*, which include unhelpful affective responses that may hinder their ability to support their child in the moment. Finally, *recovery/behavior coaching* involves teaching parents behavioral strategies to interrupt their child's symptomatic behavior. This manualization ensures that the intervention is consistent across clinicians, families, and settings. Therefore, when accessing EFFT services, families will receive a standardized intervention that targets these four key areas, equipping them to respond to challenges in productive ways. There is acknowledgment and consensus in the emotionally focused therapeutic community that EFFT shares similarities with other therapeutic approaches, including Attachment‐Based Family Therapy, Dyadic Developmental Psychotherapy, and Emotionally Focused Family Therapy (Sabey et al. 2024).

One of the advantages of EFFT is the balance between manualization and adaptability, supported by a modular and component‐based design. In addition, this approach relies on both psychoeducational and experiential (e.g., process‐oriented) work. EFFT is often conducted with the whole family over multiple sessions. However, a 2‐day caregiver group intervention has been designed as a cost‐effective alternative (LaFrance‐Robinson et al. 2016; Nash et al. 2020), and clinical guidelines have been published by the developers for telehealth or virtual delivery, instantiated by clinical need arising during the pandemic (Foroughe et al. 2023). In addition to evidence among eating disorder populations, there are studies suggesting EFFT as a transdiagnostic model, where improvements are observed in caregiver self‐efficacy following intervention among families with children who have varied clinical presentations (Foroughe et al. 2019; LaFrance‐Robinson et al. 2015). EFFT is also trauma‐informed and appears to support the reduction of parental emotional blocks (Sabey et al. 2022), in addition to improvements in child symptomatology, especially for parents with a history of maltreatment (Cordeiro et al. 2022; Foroughe et al. 2019). In long‐term follow‐ups of EFFT, there is covariation in improvements among caregivers and children, where greater symptom reduction for children corresponds to longer‐lasting improvements in caregiver self‐efficacy (Foroughe et al. 2023). There remain no published randomized controlled trials of EFFT, though at least one is underway (Thomassin 2024). Most of the aforementioned studies are single‐group clinical cohort designs, though there are also case studies published, as well (Smith et al. 2018). No research has investigated how PC changes in the context of this intervention, and there have been no larger studies of the virtual format.

## 3 The Current Study

The present study sought to investigate the 12‐month trajectories of PC following a virtual, 2‐day, caregiver group EFFT intervention completed during the COVID‐19 pandemic. Given that one family member's mental health challenges often affect the whole family system (Browne et al. 2015), we explored PC for caregivers and siblings in addition to the child with the mental health concern. Research questions included: (1) How much variance in PC is attributed to (a) change over time, (b) individual differences, and (c) family differences? (2) On average, how does PC change over 12 months following a 2‐day EFFT intervention? (3) Do children and caregivers differ in baseline levels and trajectories of PC? (4) Does caregiver social support and family dysfunction predict change in PC?

We hypothesized that there would be significant variance in PC at the family, individual, and observation levels: some families would report higher levels of PC than other families, within families, some members would have higher levels than other members, and PC would change over time, within persons. Second, we predicted that, on average, PC would increase significantly over time. Third, we hypothesized that caregivers and children would exhibit different trajectories, with caregivers showing more immediate improvements since they participated directly in the intervention. Finally, we expected that families with lower social support and higher family dysfunction would have lower levels of PC at baseline and would experience less of an increase in PC over time.

## 4 Methods

### 4.1 Participants and Procedure

The current pre‐registered study (https://osf.io/vsgk9) followed caregivers immediately before and for 12 months after a virtual 2‐day caregiver group EFFT intervention. Each workshop included approximately 25 to 30 participants and included two 7‐h training days. All caregivers attended both days for the period of the research study. Workshop activities included seminar‐style psychoeducation with personal and professional anecdotes, brief video presentations, and experiential exercises to help caregivers practice being a behavior and emotion coach for their child. Therapeutic interventions such as validating caregiver challenges, experiential skills practice, and emotion‐focused chair work to process emotion “blocks” were also offered to caregivers. These interventions were held in small groups (2–3 parents) for greater comfort and privacy and the opportunity for repeated practice. Caregivers were guided in providing emotional support to one another, while avoiding making judgments or jumping to a solution‐focused approach. Co‐facilitators monitored and supported small group exercises to ensure appropriate support was provided and to increase intervention fidelity. Day 1 focused on providing emotional support to their loved one, while Day 2's focus was primarily on behavioral support, including limit settings. On the afternoon of Day 2, emotion and behavior support were integrated, and caregivers were supported in working through any remaining fears and emotion blocks that could interfere with their desired parenting approaches.

The intervention and study were conducted through a partnership between a major research university and a large private clinic located in a major metropolitan Canadian city. Ethics approval for the current study was obtained through the University of Waterloo's ethics review board. Workshops were advertised broadly on social media and various listservs. Families were referred for the intervention by service providers (e.g., pediatricians, schools, clinicians, etc.) or by self‐referral. Given that EFFT is a transdiagnostic approach, caregivers were eligible to participate if they perceived that their child had any type of mental health challenge. Caregivers who signed up for the intervention were asked if they wanted to participate in the research study. Caregivers provided informed consent verbally over the phone and in writing before the intervention. The caregiver EFFT interventions were delivered six times a year between September 2020 and June 2022. Clinical work was led by a clinical psychologist who was certified as an EFFT trainer by EFFT's cofounders. The interventions were held consistent across groups in terms of instructor, format, content, materials, and handouts. Caregivers (76.2% married/common‐law) completed self‐ and parent‐report questionnaires at six time points: pre‐intervention (T0; *n* = 155), post‐intervention (T1; *n* = 147; 94.8%), 1‐month follow‐up (T2; 145; 93.5%), 3‐month follow‐up (T3; 142; 91.6%), 6‐month follow‐up (T4; 134; 86.5%), and 12‐month follow‐up (T5; 133; 85.8%). Caregivers completed these measures to report on themselves and up to four of their children (*M* = 2.09 children per family, SD = 0.77) between the ages of 5 and 24 years. Of children, 91.7% lived full‐ or part‐time (shared custody) with their caregiver.

### 4.2 Measures

#### 4.2.1 Child and Caregiver Positive Coping

The 10‐item Connor‐Davidson Resilience Scale (CD‐RISC‐10; Campbell‐Sills and Stein 2007) was used to measure caregiver‐reported child and caregiver PC. An example item is “I am/My child is able to adapt when changes occur” (Campbell‐Sills and Stein 2007). Caregivers responded to these statements on a five‐point Likert scale from 0 = “Not true at all” to 4 = “True nearly all the time.” Possible scores ranged from 0, indicating low PC, to 40, indicating high PC. The internal consistency of this measure was good to excellent (0.87 to 0.98).

#### 4.2.2 Caregiver Social Support

The Canadian National Longitudinal Survey of Children and Youth adapted three items from the Social Provisions Scale (Orpana et al. 2019) to create a measure of caregivers' perceived social support at baseline. An example item includes: “There are people I can count on in an emergency” (Orpana et al. 2019). These items were rated on a four‐point Likert scale with options ranging from 1 = “Strongly agree” to 4 = “Strongly disagree.” Scores range from 3 to 12, with higher scores indicating higher levels of perceived social support. The internal consistency of this measure was moderate (0.60).

#### 4.2.3 Family Dysfunction

The 6‐item general functioning subscale of the Family Assessment Device (FAD‐GF6+; Boterhoven de Haan et al. 2015) was used to measure family dysfunction at baseline. Participants rated six items on a four‐point Likert scale from “Strongly agree” to “Strongly disagree.” A sample reverse‐coded item includes “We confide in each other” (Boterhoven de Haan et al. 2015). Possible average scores ranged from 1 to 4, with scores above 2 indicating clinical impairment. The internal consistency was excellent (0.90).

#### 4.2.4 Covariates

Analyses controlled for family income, caregiver relationship status (1 = married/common‐law, 0 = else), number of children, and caregiver and child age and gender. Dummy variables were created to encompass the gender identity options: (1) in the gender‐man dummy variable, boys/men were coded as 1 (else = 0), and (2) in the gender‐minority dummy variable, gender minorities were coded as 1 (else = 0), making girls/women the reference category.

The COVID‐19 Family Stressor Scale was used to assess caregivers' experience of stressors related to the pandemic, income, and family at baseline (Prime et al. 2021). A sample item includes: “job disruption or loss.” Items were rated on a three‐point Likert scale from “Not true” to “Very true,” with higher scores indicating greater stress. The internal consistency was good (0.80).

### 4.3 Statistical Analyses

The current study used higher‐order growth curve modelling, where repeated measures were nested within individuals, who were nested within caregivers. We tested models, which were estimated with restricted maximum likelihood and compared using the likelihood ratio test. Profile likelihood was used to estimate confidence intervals and *p* values. Time was measured in months and centered on the first measurement, which occurred 1 week before the intervention. Outliers for the study variables were winsorized. Analyses were conducted in R‐Studio, using the *nlme* package (Pinheiro et al. 2023). Missing data was dealt with through multiple imputation (five imputation sets and 20 iterations), and pooled results are reported. The final sample included 155 families (level 3), 449 individuals (level 2), and 2641 observations (level 1).

From a null model, variance partitioning coefficients identified the proportion of variance in PC due to family differences (caregivers reporting on themselves and their children; level 3), individual differences (level 2), and change over time (level 1). Next, we fit an unconditional growth model to explore the average linear and quadratic change in PC over time for all participants. Random slopes (permitting separate trajectories for families and individuals within families) were added. We included the main effect and interaction with time for a dummy variable that categorized participants as a child (0) or caregiver (1). In the final model, we added covariates and predictor variables. Two‐way interactions between predictor variables and time were added as well. Interactions between gender‐man and age with “caregiver” were also included to investigate whether the association between gender and age with PC differed for children and caregivers. Gender‐minority was not added as an interaction due to small cell size. COVID stressors were added as a covariate as an exploratory analysis. Family dysfunction, social support, COVID stressors, and income were grand‐mean centered.

#### 4.3.1 Sensitivity Analyses

Of participating families, 20% included more than one caregiver from the same household (i.e., both partners in a family participated in the intervention and study). Therefore, some caregiver dyads reported on the same children. Sensitivity analyses were conducted to compare the intraclass correlations (ICCs) between the samples with and without these families. The ICCs did not differ between samples; therefore, the study included all responses. We also conducted sensitivity analyses to explore (1) whether the number of caregivers per family who participated in the intervention was associated with differences in trajectories and (2) whether PC differed between the child with the mental health concern (the target child) and their sibling(s). Results are reported below.

## 5 Results

Table 1 presents the descriptive statistics and bivariate correlations between variables.

**Table 1:** Descriptive statistics and bivariate correlations for the study variables and covariates.

Variable	1	2	3	*M*	SD	Range
1. Age	—					
(a) Child				12.24	3.82	5–24
(b) Caregiver				46.78	7.03	30–78
2. Family dysfunction	−0.012	—		2.06	0.59	1–4
3. Social support	0.043*	−0.365*	—	10.11	1.57	6–12
4. Positive coping	0.314*	0.231*	0.176*	24.41	7.66	0–40
Gender	Caregiver *n* (%)	Child *n* (%)	All *n* (%)			
a) Girl/woman	116 (74.8%)	155 (53.3%)	271 (60.8%)			
b) Boy/man	38 (24.5%)	124 (42.6%)	162 (36.3%)			
c) Gender minority	1 (0.6%)	12 (4.1%)	13 (2.9%)			
Ethnicity						
a) White	111 (71.6%)	210 (72.2%)	321 (72.0%)			
b) South Asian	16 (10.3%)	29 (10.0%)	45 (10.1%)			
c) East Asian	13 (8.4%)	26 (8.9%)	39 (8.7%)			
d) Black	5 (3.2%)	15 (5.2%)	20 (4.5%)			
e) Indigenous	3 (1.9%)	10 (3.4%)	13 (2.9%)			
f) Hispanic	3 (1.9%)	8 (2.7%)	11 (2.5%)			
g) Middle Eastern	1 (0.6%)	6 (2.1%)	7 (1.6%)			
h) Pacific Islander	1 (0.6%)	3 (1.0%)	4 (1.0%)			
i) West Indian	1 (0.6%)	1 (0.3%)	2 (0.4%)			
j) Self‐described	10 (6.5%)	37 (12.7%)	47 (10.5%)			
Income						
a) $0–1600			3 (1.9%)			
b) $1601–3300			8 (5.2%)			
c) $3301–5000			18 (11.6%)			
d) $5001–6600			16 (10.3%)			
e) $6601–8300			28 (18.1%)			
f) $8300+			82 (52.9%)			

*Note: M* = mean/average; SD = standard deviation, *n* = number of participants.

* *p* < 0.05, or a “statistically significant difference.” Positive coping is averaged across all participants.

### 5.1 Question 1: Variance Partitioning

**Figure 1:**
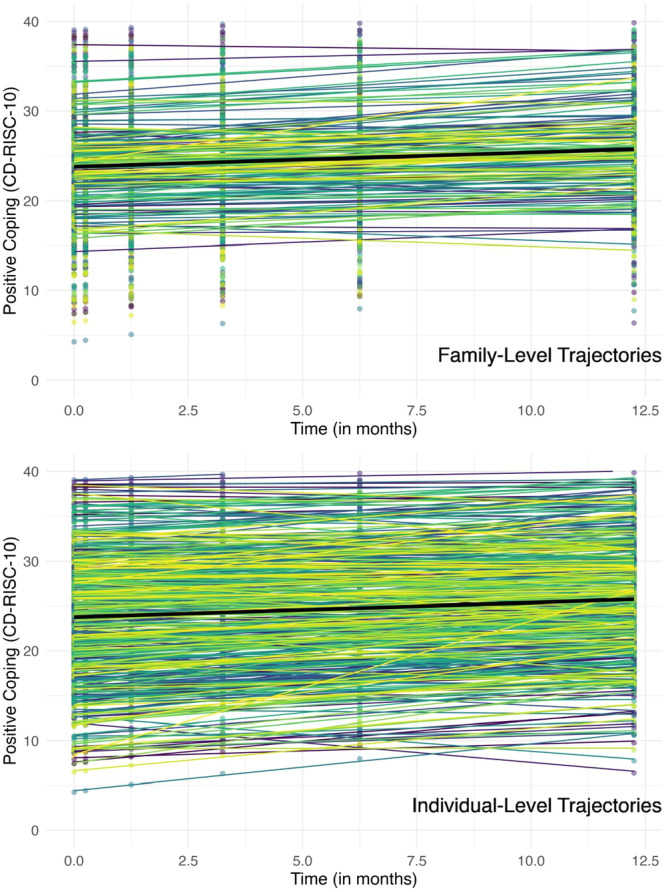
Family‐level and Individual‐level trajectories of positive coping. *Note:* The dots represent individual participants at each time point. Each colored line in the top graph represents a predicted family trajectory, and each colored line in the bottom graph represents a predicted individual trajectory. The black lines (in both top and bottom graphs) represent the mean predicted trajectory for all participants.

The intraclass correlations revealed significant between‐family, between‐individual, and within‐individual differences in PC. In the null model, 15.8% of the variance in PC was due to family differences, 55.0% was due to individual differences, and 29.2% was due to change over time and measurement error. The significant between‐family variance revealed that individuals within the same family are more similar in terms of their PC scores compared to participants from other families. However, the significant and sizable between‐individual variance indicates that there are major differences among individuals within the same family in terms of their PC. Finally, the significant within‐individual variance indicates that an individual's PC score changes over time (and there is measurement error). These three levels are displayed in Figure 1. The relative proportion of variances highlights the importance of measuring PC across individuals within the same family.

### 5.2 Question 2: Unconditional Growth Model

**Figure 2:**
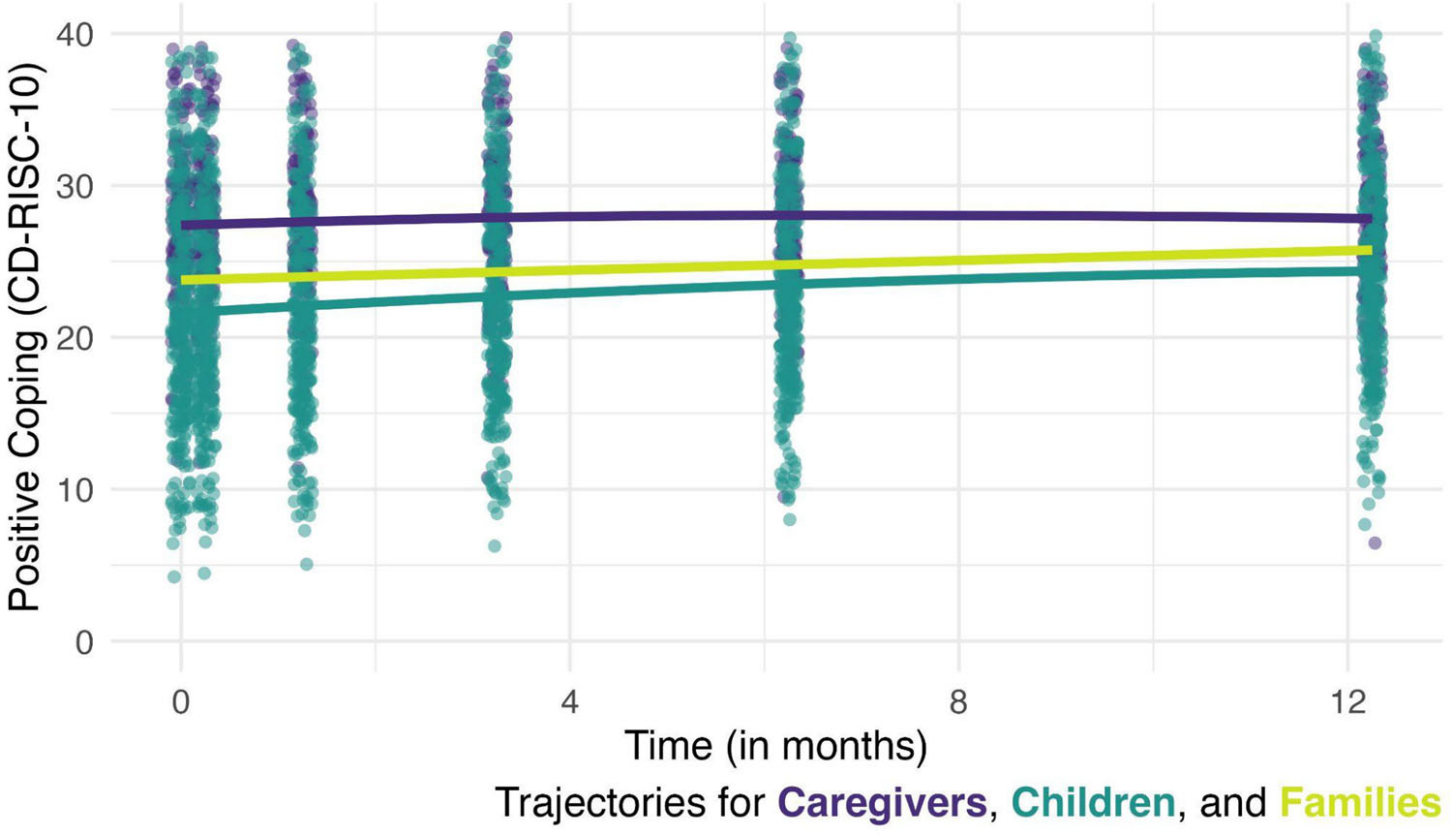
Trajectories of positive coping for all participants, caregivers, and children. *Note:* The teal dots represent individual children, and the purple dots represent individual caregivers at each timepoint. The teal line represents the mean predicted trajectory for all children, the purple line represents the mean predicted trajectory for all caregivers, and the light green line represents the mean predicted trajectory for families (all participants).

Tables 2 and 3 and Figure 2 display the growth curve models. In the random intercepts model (Model 2), participants started with PC levels at *b* = 23.78 (95% CI = 23.07, 24.48) and significantly improved over time (*b* = 0.17, 95% CI = 0.14, 0.20). In the random slopes model (Model 3), random effects reveal that families differ in their linear trajectories and that individuals within families differ in their trajectories relative to the family average trajectory. Moreover, within families, the significant covariance between the individual‐level random intercept and slope (*b* = −0.54, 95% CI = −0.66, −0.40) indicates that individual members with lower initial levels of PC experienced greater increases in PC over time. The intercept/slope covariance at the family level led to convergence issues; therefore, the covariance was constrained to zero. Model 4 explored the quadratic trajectory of PC, which was also significant (*b* = −0.01, 95% CI = −0.02, −0.01). Participants displayed a rapid initial increase in PC after the intervention, and this rate of improvement decreased over time.

### 5.3 Question 3: Caregiver and Child Differences

Model 5 considered caregiver and child differences. There were significant differences in PC at baseline, with caregivers having higher PC compared to children (*b* = 5.89, 95% CI = 4.63, 7.16). In addition, caregivers and children differed in their trajectories, with caregivers displaying less change over time (*b* = −0.19, 95% CI = −0.26, −0.11), though their improvement was still statistically significant.

### 5.4 Question 4: Covariates and Predictor Variables

In Model 6, covariates and predictors were added. Gender‐minority, boy/man, age, income, and relationship status did not significantly predict initial values of PC. However, the caregiver*boy/man interaction was significant (*b* = 3.10, 95% CI = 0.44, 5.76), whereby participants who identified as a man/boy scored higher in PC if they were caregivers, but lower in PC if they were children. Caregivers with more children reported higher baseline PC for themselves and their children (*b* = 0.87, 95% CI = 0.06, 1.60). COVID stressors significantly predicted baseline PC (*b* = −0.16, 95% CI = −0.28, −0.04), such that higher stressors were associated with lower PC. As an exploratory analysis, the COVID stressors*months and income*months interactions were explored, which were nonsignificant. Social support predicted PC at baseline (*b* = 0.52, 95% CI = 0.06, 0.98), whereby caregivers with higher social support reported higher PC in themselves and their children. Family dysfunction also predicted PC at baseline (*b* = −1.89, 95% CI = −3.16, −0.63), whereby caregivers who reported greater dysfunction reported lower PC in themselves and their children. Social support and family dysfunction did not affect trajectories of PC.

### 5.5 Sensitivity Analyses

There were no differences in trajectories of PC for families with one versus two caregivers who participated in the intervention (*b* = −0.05, 95% CI = −0.18, 0.08). Results revealed that target children had lower PC than siblings (*b* = −9.21, 95% CI = −10.37, −8.04) and caregivers had higher PC than siblings (*b* = 1.20, 95% CI = 0.05, 2.35). Furthermore, when exploring trajectories, target children improved more quickly than siblings (*b* = 0.28, 95% CI = 0.19, 0.36), and siblings and caregivers did not differ in their rate of improvement (*b* = −0.04, 95% CI = −0.13, 0.04).

**Table 2:** Fixed effects of the multilevel model output examining family and individual trajectories and positive coping.

	*B* [95% CI]					
Fixed effects	Model 1 (Null)	Model 2 (Random intercept)	Model 3 (Random slopes)	Model 4 (Quadratic)	Model 5 (Caregiver)	Model 6 (Predictors)
Intercept	24.43* [23.73, 25.12]	23.78* [23.07, 24.48]	23.78* [23.02, 24.53]	23.60* [22.84, 24.35]	21.48* [20.61, 22.35]	21.99* [18.67, 25.32]
Months		0.17* [0.14, 0.20]	0.17* [0.12, 0.21]	0.33* [0.22, 0.44]	0.40* [0.29, 0.51]	0.40* [0.29, 0.51]
Months^2				−0.01* [−0.02, −0.01]	−0.01* [−0.02, −0.01]	−0.01* [−0.02, −0.01]
Caregiver					5.89* [4.63, 7.16]	1.45 [−5.39, 8.29]
Boy/man						−0.89 [−2.32, 0.55]
Gender minority						−1.31 [−4.83, 2.22]
Age						−0.12 [−0.31, 0.08]
Number of children						0.87* [0.06, 1.69]
Married/common‐law						−0.81 [−2.41, 0.80]
Income						0.19 [−0.32, 0.71]
COVID stressors						−0.16* [−0.28, −0.04]
Family dysfunction						−1.89* [−3.16, −0.63]
Social support						0.52* [0.06, 0.98]
Caregiver*Months					−0.19* [−0.26, −0.11]	−0.19* [−0.27, −0.12]
Caregiver*Boy/man						3.10* [0.44, 5.76]
Caregiver*Age						0.16 [−0.06, 0.39]
Months*Family Dysfunction						−0.02 [−0.10, 0.07]
Months*Social Support						−0.01 [−0.04, 0.02]

*Note:* Family dysfunction, social support, COVID stressors, and income were centered at their grand mean. 95% CI = 95% confidence intervals; *B* = unstandardized estimates.

* *p* < 0.05, or a “statistically significant difference.”

**Table 3:** Random effects and model fit statistics of the multilevel model output examining family and individual trajectories and positive coping.

	SD [95% CI]					
Random effects	Model 1 (Null)	Model 2 (Random intercept)	Model 3 (Random slope)	Model 4 (Quadratic)	Model 5 (Caregiver)	Model 6 (Predictors)
Level 3						
Random Intercept	1.88* [1.00, 3.53]	1.88* [0.99, 3.56]	2.18* [1.31, 3.60]	2.18* [1.31, 3.62]	2.64* [1.88, 3.69]	1.63* [0.78, 3.37]
Random Slope			0.17* [0.13, 0.23]	0.17* [0.13, 0.23]	0.16* [0.12, 0.22]	0.17* [0.13, 0.23]
Level 2						
Random Intercept	6.56* [6.04, 7.12]	6.57* [6.05, 7.13]	6.91* [6.40, 7.46]	6.91* [6.40, 7.47]	6.18* [5.70, 6.69]	6.10* [5.63, 6.61]
Random Slope			0.26* [0.22, 0.31]	0.26* [0.22, 0.31]	0.25* [0.21, 0.30]	0.25* [0.20, 0.29]
Cov (Intercept/Months)			−0.54* [−0.66, −0.40]	−0.54* [−0.66, −0.40]	−0.47* [−0.60, −0.32]	−0.45* [−0.58, −0.29]
Level 1						
Random Intercept	3.49* [3.39, 3.59]	3.40* [3.30, 3.50]	3.09* [2.99, 3.19]	3.08* [2.98, 3.18]	3.08* [2.98, 3.18]	3.08* [2.98, 3.18]
*Model fit*						
AIC	15702.12	15593.24	15460.20	15460.03	15391.32	15380.20
BIC	15725.69	15622.70	15507.33	15513.05	15456.11	15521.45
LogLike	−7847.06	−7791.62	−7722.10	−7721.01	−7684.66	−7666.10
*L* Ratio		110.88	139.05	2.17	72.71	37.12
df	4	5	8	9	11	24
*p*		< 0.001	< 0.001	0.141	< 0.001	0.001

Abbreviations: 95% CI, 95% confidence intervals; AIC, akaike information criterion; BIC, Bayesian information criterion; Cov, covariance; df, degrees of freedom; LogLike, log‐likelihood; L Ratio, likelihood ratio test; SD, standard deviation.

* *p* < 0.05, or a statistically significant difference.

## 6 Discussion

This study aimed to explore the extent to which PC was a characteristic of families, individuals, and time, in addition to how PC changed over 12 months following a 2‐day caregiver EFFT intervention, along with the predictors of this change. Our first three hypotheses were supported: (1) there was a significant amount of variance in PC at the family, individual, and temporal levels, (2) participants displayed an increase in PC over time pre‐post‐intervention (though the rate of improvement attenuated over time), and (3) the average trajectories differed for children and caregivers. Our final hypothesis was partly supported: while family dysfunction and social support predicted baseline PC, they did not predict rates of change.

### 6.1 Positive Coping as a Family‐Level Phenomenon

Consistent with our first hypothesis, a significant proportion of the variance in PC was explained by family‐level differences, individual differences, and change over time (and measurement error), suggesting that PC may be a shared family phenomenon, an individual psychological trait, and a person‐context process. The significant family‐level differences suggest that there are likely family‐wide factors that promote or impede PC. Individuals within the same family generally share genes, and genetic factors are associated with PC (Dunn and Conley 2015; Navrady et al. 2018). While the current study accounted for family clustering, which likely correlates with genetic relatedness, our design cannot draw genetic conclusions.

Irrespective of genetics, families often have shared environments that may similarly influence family members. In the present study, social support and family dysfunction were family‐level factors that correlated with PC. However, these constructs were solely reported on by caregivers, potentially contributing to these associations. Notwithstanding, this result is consistent with research and theoretical models on coping (Brincks et al. 2010; Cong et al. 2020; DeLongis and Holtzman 2005; Masarik and Conger 2017; McCubbin and Patterson 1983; Ren et al. 2018; Ye et al. 2020). The Double ABC‐X Model outlines the promotive relationship of family resources, such as social support and family functioning, on family coping and adaptation (McCubbin and Patterson 1983). Masarik and Conger (2017) expand on this relationship by suggesting that social support and family functioning can act as protective factors in times of stress.

There are various putative mechanisms underlying these relationships. First, individuals with higher social support likely receive instrumental and emotional support, which can help them overcome challenges (Walsh 2016). Additionally, this support contributes to more positive caregiver mental health (Letvak 2002), parenting and parent–child relationships (Letourneau et al. 2001), and family functioning (Sloss et al. 2024), which can positively impact both caregivers and children. Simultaneously, the mechanisms underlying how dysfunctional family environments can hinder PC are multifaceted. For example, hostile interactions are associated with greater physiological and emotional reactivity and lower self‐regulation (Hosseini‐Kamkar et al. 2021), leading individuals to react to stressors in ways that do not align with their current goals. Attachment processes may also play a role (Camisasca et al. 2017), whereby insecurely attached individuals may learn that relationships are not always supportive or stable, and therefore, may not reach out to others for support. In response to the uncontrollability of family conflict, some people respond with avoidant or placating behaviors (Repetti et al. 2002), which are responses that may prevent them from taking action to address the situation or advocate for their needs. Lastly, Ren and colleagues (2018) recognize that in dysfunctional environments, children may see adults modelling negative coping, leading them to adopt similar strategies.

Surprisingly, baseline family dysfunction and social support did not predict the rate of change in PC. In other words, patterns of change in PC over 12 months (which showed an overall increase) were experienced by families with high and low levels of these risk and protective factors. It is possible that these factors did not further affect change or that change in PC processes occurred within individual dyads in the family system (e.g., between a parent and their child) and were less discernible at the whole family level. Alternatively, the intervention may have been associated with increases in caregiver social support and family functioning. The intervention's emotion coaching and therapeutic apologies modules may have contributed to more positive familial relationships and family processes. In addition, as the family context became healthier, caregivers may have had more ability to nurture relationships outside of the family unit. As a result, caregivers may have reported higher social support and family functioning in follow‐up measures. Consequently, baseline levels of these family resources may not have accurately predicted changes in PC.

The association of family‐level factors and PC highlights the importance of considering the family context in clinical interventions. In individual therapy, therapists and clients often consider how the family contexts may have played a role in the current challenges, including how parent mental health and family‐wide stressors relate to parenting and child outcomes. In family therapies, the whole family is often involved in treatment. Family members may learn how their shared family experiences may be contributing to the presenting problems. In response, they can work on reducing unhealthy interactions and learn strategies to develop a supportive family environment that fosters well‐being for all members.

EFFT is an intervention that involves families because of the role that family members play in a child's challenges (Foroughe et al. 2019; LaFrance‐Robinson et al. 2015). As previously described, EFFT supports caregivers in four areas, which may foster positive child coping. Focusing on repairing the parent–child relationship may increase trust and security. Consequently, children may seek support from their caregiver when experiencing distress. Additionally, when caregivers engage in emotion coaching, they enable their child to face, rather than avoid, their emotions, which is an important aspect of effective coping. Finally, teaching caregivers behavioral skills and helping them address their “emotional blocks” enables them to respond more effectively to their children's challenges, encouraging PC.

While parenting and family interventions can be effective, they may be limited if families are experiencing risk factors, such as poverty (Zilberstein 2016). Interventions that fail to account for social disadvantage may not be relevant to these families. In the current study, income did not predict change in PC; however, this interaction may be significant in a sample with greater income variation. Therefore, interventions should seek to understand each family's unique resources and stressors. Furthermore, a high degree of stressors may overwhelm a family's ability to cope, so they may not have the capacity to focus on changing their family interactions. Instead, multilevel interventions that include systemic policy changes addressing pressing social issues (e.g., poverty, food/housing insecurity, displacement, forced migration, incarceration, child welfare, etc.) may be necessary for these family interventions to be effective for the highest‐risk families (Browne et al. 2024).

### 6.2 Individual‐Specific Influences in Positive Coping

In addition to family‐level factors, variability in PC was influenced to the greatest extent by individual differences. In the current study, caregivers who were men, and children who were girls, had higher PC. Eschenbeck et al. (2007) found that girls tended to cope through problem‐solving and seeking support, whereas boys tended to cope through avoidance, potentially because of gender biases in how adults encourage children to express emotions (Amin et al. 2018). In contrast, various studies have found that women tend to have lower CD‐RISC‐10 scores than men (Kavčič et al. 2023; Pulido‐Martos et al. 2020; Smith et al. 2018). This finding may be related to gender differences in the specific items of the measure. For instance, the CD‐RISC‐10 focuses on aspects of coping that are more common in men (e.g., personal competence and internal control), and less on aspects of coping more common in women (e.g., support‐seeking and spirituality; Kavčič et al. 2023). Future research should investigate additional individual‐level factors that may affect PC.

### 6.3 Caregiver and Child Differences

When comparing children and caregivers, caregivers perceived that they had higher PC than their children. However, sensitivity analyses revealed that target children were rated as significantly lower than siblings, who were rated as significantly lower than caregivers in PC at baseline. It is not surprising that target children had the poorest PC given the relationship between coping style and mental health (Gurvich et al. 2021). In terms of the differences between children (target children and siblings) and caregivers, it is possible that children were more negatively impacted by the COVID‐19 restrictions than caregivers (Runacres et al. 2021). The study controlled for caregiver‐perceived COVID stressors at baseline; however, caregivers may have differed from children in their perception of these stressors. There could also be generational differences that affect PC, including the use of digital media, which has been increasing drastically over the last few decades, and was exacerbated by the pandemic (Paschke et al. 2021). This rise in media use is associated with lower attention spans (Jourdren et al. 2023), which may negatively impact persistence when encountering challenges (Akomolafe and Ajayi 2019). Future studies could investigate whether media use is associated with PC. Finally, caregivers completed the measures for themselves and their children, which may have contributed to social desirability bias.

Caregivers and children also differed in PC trajectories. When comparing children and caregivers, children displayed marked improvement in PC over time, increasing at a faster rate than caregivers, who exhibited a modest, yet still significant, improvement. However, the sensitivity analyses revealed that target children improved the most and that siblings and caregivers did not significantly differ in rate of change. There are a few possible explanations for these differences. First, parents and siblings had higher baseline PC than target children, suggesting that target children may have been poised to benefit more in this area. Additionally, the intervention focused on improving parenting and parent–child interactions—especially with the target child—which may have led to greater improvements in PC for target children, compared to their caregivers and siblings.

### 6.4 Limitations and Future Directions

The current study had several limitations. First, all variables were assessed by caregiver self‐ and parent‐report measures, which may have contributed to social desirability and shared method variance bias. Future studies should employ more objective measures (e.g., observation) and self‐report measures for all family members, where possible, based on age considerations. Second, the sample included moderate ethnic and income diversity, which may limit generalizability. In the future, studies should aim to recruit a sample that is more representative of the general population. Furthermore, the same clinician administered the workshops, which may limit the generalizability to workshops led by different clinicians. Third, the predictor variables and covariates were time‐invariant. Consequently, the study did not consider how changes in these variables may have been associated with PC. We did not explore changes in COVID stressors, which likely fluctuated since restrictions would have changed over the 12 months of data collection. Additionally, the study only included limited individual‐level predictor variables, which did not allow for an in‐depth investigation of the factors that may have contributed to the substantial individual‐level variance in PC. Future studies should include time‐variant and more varied individual‐level predictor variables. In addition, the caregiver social support scale had moderate internal consistency. Lastly, while it is hypothesized that the EFFT intervention may have contributed to the observed improvements in PC, causation cannot be determined, highlighting the importance of the randomized control trial currently conducted by Thomassin (2024).

## 7 Conclusion

The present study found that family differences, individual differences, and time explained a significant proportion of variance in PC. Additionally, participants displayed an overall increase in PC over the 12 months following a brief EFFT intervention. Caregivers and children differed, with caregivers reporting higher baseline PC, but a slower improvement rate than children. Finally, family dysfunction and social support were associated with lower and higher baseline PC, respectively; however, these variables did not significantly influence change. These findings reveal that PC can improve over time and that family‐level factors play a significant role in shaping PC. Therefore, the family environment should be considered when engaging in interventions promoting PC. In addition to supporting PC as an intrapsychic phenomenon, whenever possible, interventions might aim to include family members to comprehensively support family‐wide PC across the developmental ecology.
